# Distributed implantable microchips as wireless networks for neural sensing and stimulation

**DOI:** 10.3389/fnins.2026.1855768

**Published:** 2026-09-28

**Authors:** Ah-Hyoung Lee, Jihun Lee, Vincent Leung, Lawrence Larson, Arto Nurmikko

**Affiliations:** 1 Department of Robotics and Mechanics Engineering, Daegu Gyeongbuk Institute of Science and Technology, Daegu, Republic of Korea; 2 Department of Brain and Cognitive Sciences, Korean Advanced Institute of Science and Technology, Daejon, Republic of Korea; 3 Electrical and Computer Engineering, Baylor University, Waco, TX, United States; 4 School of Engineering, Brown University, Providence, RI, United States

**Keywords:** brain-computer interfaces, neural decoding, neural sensors, neuroengineering, wireless brain implants, wireless communication

## Abstract

We examine ongoing research for implantable sensors and stimulators for future brain-computer applications and give a review on the concept where grain-of-sand size silicon microchips form the individual nodes in a multichannel wireless implant. In such an approach, each chip can perform the function of a stand-alone independent sensor if equipped with active microelectronic circuits designed to detect or stimulate target populations of neurons and capability for wireless communication. Delivering power to individual as well as spatially distributed ensembles of microchips requires a method for efficient transcutaneous/transcranial wireless energy transfer approach. Here we offer examples from authors’ experience and comparisons to contemporary approaches as well as the challenges ahead in developing clinically viable wireless cortical chip-in-the-brain interfaces.

## Introduction

Research to advance brain-computer (BCI) technology has seen significant progress from the first demonstrations in clinical pilot trials of paralyzed persons controlling computer cursor and related communication-enabling neuromotor prostheses (Hochberg et al., 2006; Afshar et al., 2011; Aflalo et al., 2015; Pandarinath et al., 2017). Recent advances have extended the use of multielectrode-based recording to more complex tasks such as in brain-to-voice BCIs (Littlejohn et al., 2025, Willett et al., 2023) as well shown the possibility of using multielectrode intracortical stimulation to evoke percepts such as for tactile substitution (Valle et al., 2025). The state of clinical trials of present BCI technology has been subject to recent reviews (Patrick-Krueger et al., 2025). The pioneering early work also beckons new neuroengineering opportunities in developing implants to access much larger populations of neurons than allowed by present probes.

The first generation, monolithic silicon microelectrode cortical sensor arrays (MEAs) were developed twenty plus years ago, notably the ‘Utah’ or ‘Michigan’ intracortical sensors (Normann et al., 1999; Najafi et al., 1985). These electrically passive devices have a steady workhorse in BCI work, as have any number of rigid or flexible epicortical (ECoG) configurations for recording field potential neural population signals. Increasing the channel count and thus the clinical scalability, for example, of the Utah MEA is challenging because of the complexity of transcranial wiring and surgical considerations. Still, as many as 16 intracortical microelectrode arrays were implanted into a non-human primate (Chen et al., 2020) to access approximately 1,000 channels of recording and stimulation across the visual cortex on a square centimeter scale.

Scaling up current sensors and actuator platforms to access up to thousands of circuit points across the cortex for human clinical use is an engineering opportunity as well as a challenge for next generation of high-performance brain-computer interfaces. Biocompatibility, viable surgical insertion, and management of ever-increasing neural data flow are some key considerations. Examples of large scale sensor designs include intracortical probes such as the ultrathin silicon stiletto design of Neuropixel probes (Jun et al., 2017), dense multielectrode threads inserted robotically to the cortex by Neuralink (Musk and Neuralink, 2019), ultradense microwire arrays by Paradromics (Obaid et al., 2020), polymer-based stereo-electrode arrays (Lee K. et al., 2024), as well as a number of dense ECoG micro arrays designs for epicortical recording of field potentials such as by CorTec (Cho et al., 2024); note also recent review (Xie et al., 2024).

Another goal for BCIs is to develop a mobile neurotechnology whereby data from/to implanted neural probes is transmitted wirelessly outside the body. There are in principle two approaches to configuring a wireless interface in the context of monolithic cortical sensors implants. In one approach, intracortical probes are connected by a short transcranial lead to a wireless, subdermal, hermetically sealed RF transceiver package. This has been demonstrated in non-human primate research (Borton et al., 2013, 2016) and in a related scheme with the wireless module slotted to the skull deployed in human subjects at Neuralink (Musk and Neuralink, 2019). Full integration of active electronics directly with the sensor element is challenging, namely hermetically packaging the sensor seamlessly with the adjacent microelectronic device layer. For large channel counts, the added performance requirements of low power yet sufficient data and bandwidth add constraints to electronics design. Further, wireless powering and communication protocols must conform to regulatory limits such as for RF exposure, thereby placing a premium on low power electronics (Sahasrabudhe et al., 2025). Recently, however, such integration has been shown possible in an all-on-Si-wafer approach that incorporated active electronics (Chiang et al., 2020) as well as in polymer-based scalable multielectrode arrays (Lee K. et al., 2024).

This review focuses on a new class of neural sensors, a concept in which monolithic, microelectrode devices are ‘broken up’ as a population of silicon microchips. Here each autonomous single microchip channel transmits recorded data to a common external hub so that a spatially distributed ensemble is equivalent to an implanted monolithic microelectrode probe but now allowing arbitrary placement of the individual sensor nodes across the cortex. As discussed below, embedded silicon microelectronics must be implemented as very low power ‘system-on-chip’ circuits, constrained in size to a fraction of square millimeter. Bidirectional communication involving large numbers of wireless nodes requires the development of customized bidirectional telemetry methods with low error rates and low latency. Most important, means for efficient wireless power delivery to populations of spatially distributed autonomous sensor/stimulator microimplants is a critical part of the system design, whether by electromagnetic means whether by radio frequency, ultrasound, or light as the energy modality of choice. Ultimately, considerations of regulatory safety places an upper limit to any wireless powering scheme, the key factor which imposes severe limitations and challenges in the microchip electronic circuit design and fabrication.

Outside this article but of seminal importance to the design of microelectronic chips is the downstream demands of neural signal decoding. Decoding neural data from thousands of channels individually transmitted by microdevice nodes adds a significant computational burden to the ambition of next generation BCIs. For review see Siegle and Steinmetz (2026). Computational machinery, particularly machine learning and related AI-tools may require innovative approaches to algorithmic models to decipher cortical population codes with minimal latency. Extending the capability of neural stimulation such as delivery of space–time patterned near naturalistic stimulus requires closing a computational read-out/write-in within a closed loop with minimal delays.

## Concept of spatially distributed autonomous microsensors/actuators

The concept cartoon Figure 1 for a large-scale neural sensor/actuator illustrates the approach where a fully wireless implant builds from independent, spatially distributed grains of silicon microchips. As an ensemble, the population of chips, each with bidirectional telemetry capability is effectively one wireless externally powered sensor/actuator network. Each standalone silicon microchip, a “neurograin” on or in the cortex (Lee J. et al., 2021) in the language of this paper, houses ultralow power circuits which either sense electrical signals from nearby neurons, or stimulate a small group of adjacent neurons. A number of different terms have been used for such microchip constructs, from ‘Neural Dust’ (Seo et al., 2015), to ‘Neural Motes’ (Lee et al., 2024b; Lee et al., 2025) to ‘Microbeads’ (Khalifa et al., 2019) as well as other names, all with the common aim to record and stimulate cortical circuits at cellular level spatial resolution.

**Figure 1:**
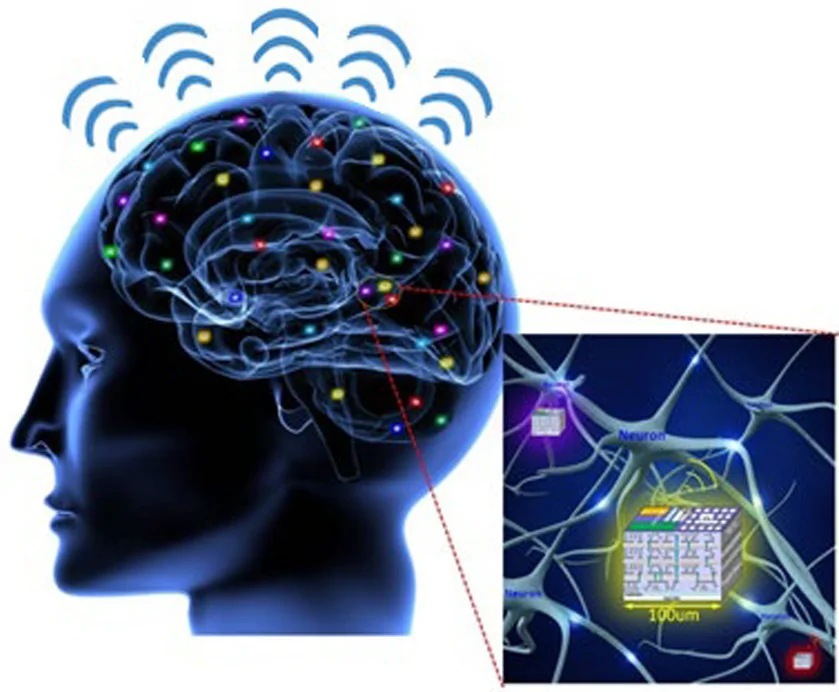
Concept cartoon for the idea of distributing a wireless network of microchip sensors and stimulators across the brain cortex.

Whether epi- or intracortical, the size of individual microchips must be small to minimize brain and tissue displacement, hence potential long-term damage. Silicon volumes on the order of 10^−3^ mm^3^ if not smaller (linear dimensions on 100 μm) are called for, adding up to mm^3^ aggregate tissue displacement for, say a thousand microchips. The anatomical and physiological constraint places a significant burden on the design of the multiple electronic functions on each neurograin on such small footprint, also influencing the choice of a particular semiconductor foundry process node.

In the sensing mode the onboard circuits are usually designed for capturing the fundamental bits of neuronal communication, namely “spikes” (action potentials) or broadband brain activity. Activity patterns recorded from potentially thousands of independent sensing points must then streamed wirelessly through the skull and the scalp to an external transceiver. From telecommunication network point of view, of importance is the on-chip design and implementation of wireless data protocols at a low cost to bandwidth and transmission at low error rates.

There are number of ways to dissect different current approaches to building a wireless system based on microchip implants of Figure 1, among which the clearest differentiator is a chosen method of wireless power delivery and the associated energy modality of choice. Methods for data transmission from/to the implant (uplink’ for sending out neural data, and ‘downlink’ for sending instructions and energy in case of stimulation) can use the same form of energy or rely on a different choice. Tissue thickness (scalp plus skull) of the target species is a key factor because of different physics of transmission losses, ranging from approximately 1 millimeter in a small rodent to more than 10 mm in primates.

## Ultrasound vs optical vs radiofrequency (RF)

The three mainstream forms of energy used so far in research demonstrations to power microchip implants in animals are ultrasound, radio frequency (RF), and light, respectively. As well as combinations thereof. There has also been innovation in the space of using external magnetic fields to power a several millimeter size neural sensors and stimulators that integrate established magnetoelectric materials such as, e.g., Metglas with electronics in one implant (Singer et al., 2020), approaches for direct inductive generation of current stimulus by microcoil implants (Lee et al., 2016), and ideas on cellular scale of local current generating magnetoelectric ‘nanodisks’ (Kim et al., 2026). The schemes of magnetoelectric conversion to electrical power depend on microfabrication and assembly of heterogenous composites of magnetic/magnetostrictive thin films with silicon.

Ultrasound approach was demonstrated as an early useful energy modality in work on ‘neural dust’ (Seo et al., 2016), both for wireless powering and detecting neural signals from single mm-size microdevices. Incident ultrasound converts to electrical power to drive silicon microcircuits for neural signal detection or stimulation (Ghanbari et al., 2019; Piech et al., 2020). Conversely, the on-chip piezoelectric crystal transduces the detected signal to modulation of ultrasound, detectable via backscattering. The method is attractive in that it benefits from the relative low tissue losses of ultrasound in the MHz frequency range, thus low thermal footprint, and is potentially scalable from the rodent models used to primates as well as application to deeper brain structures. However, bandwidth limitations may restrict the scaling of this approach to large networks of distributed ensembles devices. Individual chips have been demonstrated as implants to the peripheral nerves of small rodents but have not so far implanted in the brain or demonstrated as larger ensembles of wireless sensors.

By contrast, light-energy powered (fully optical) microimplants rely on chipscale photovoltaic elements for optical power harvesting and/or a bidirectional optical data link, particularly at near infrared wavelengths (near 900 nm) which afford somewhat longer penetration to tissue and are yet compatible with semiconductor optoelectronic technology (silicon and III-V compound semiconductors). Simplified, single optical motes of 0.002 mm^3^ which integrate microscale GaAs photovoltaic converter/optical link with CMOS circuits have been demonstrated on benchtop as well as in anesthetized rats to deliver current stimulus to the animal’s cortex and record evoked spikes (Lee et al., 2023). The functionally more versatile microelectronic circuits on-board the free floating ‘StiMote’ included designs for time-domain multiple kb/s telemetry and time domain multiplexing (TDMA), hence making the approach in principle scalable to multiple implants (Lee et al., 2024b). The chip design could include a light-tolerant version whereby optical-electrical mutual interference artifacts could be suppressed (Lim et al., 2022). Subsequently even smaller ‘nanoliter’ size single wireless optical motes employing AlGaAs diodes for dual PV and LED functions have been reported for chronic recording in awake mice (Lee et al., 2025).

While optical implants can be engineered to be considerably smaller than the ultrasound-driven Neural Dust (or comparable to Neurograins discussed below), an optical approach is fundamentally limited by the strong light scattering by tissue (scalp and skull), hence applications to larger animals especially primates, becomes exceedingly difficult because of losses.

A number of other schemes have also been reported for small-size wireless implants which represent mixed energy harvesting and telemetry modalities such as the ‘Neuroflare’ (Zhao et al., 2025) in which an inductive RF powers and reads out an on-chip LED/photodiode device intended for optical stimulation and recording. Likewise, inductive link was used for 4-channel electrical recording and optogenetic stimulation in a > 1 mm size chip design (Ramezani et al., 2018).

## RF-based approaches to microchip implants

As seen from Table 1, the benefits and disadvantages of microchip implants based fully on RF energy harvesting and RF signal transmission lie roughly between ultrasound and optical approaches particularly when considering a wireless neural interface for large animal models and primates with an order of magnitude thicker skull plus scalp (~ 1 mm in rat to ~ 10 mm in primates). This goal is yet to be met by RF-powered “neurograins” in extending current work from rodents to primates - as well as other wireless microchip implants. Still, an all-RF approach does offer the simplicity of all-silicon monolithic solution for a large-scale cortical implant spanning potentially several cm2 of cortical area - without the need for heterogeneous material integration such as in ultrasound or optical case. Importantly, designing communication protocols for a silicon microelectronic implant can take advantage of methods from wireless telecommunication technologies, regardless of whether the implant is a single ultrathin, large area silicon wafer (Hettick et al., 2026) or distributed, autonomous, microchips considered in this article.

**Table 1:** A qualitative comparison of the benefits and disadvantages of the three principal energy modalities as examples of the current generation of microchip implants.

	Neural Dust (Seo et al.)	Neurograin (Lee J et al.)	Optoelectronic mote (Letner et al.)
Implant concept	Distributed chips	Distributed chips	Distributed Chips
Chip volume	2.4 mm^3^	0.01 mm^3^	0.02 mm^3^ (w/o electrodes)
Energy harvesting	ultrasound	RF	NIR photovoltaic
Communication	ultrasound	RF	NIR (active LED)
Neural recording demo	Yes (nerve muscle)	Yes (rat cortex)	Yes (rat cortex)
Brain recording	No	Yes	Yes
Multiple functional devices	No	Yes	No
Individually addressable network	No	Yes	No
Tissue penetration	Deep (> cm)	Short (~1 cm with relay)	Very short (~ 1 mm)
Heterogeneous material integration	Yes	No	Yes

Table includes an example of the all-RF techniques (under appellation ‘neurograins’) which we turn to next.

Below we first visit key circuit considerations for wireless microchip implants before focusing on methods of RF energy harvesting and telecommunication protocols for large-scale recording implants, respectively. Review of recent work on chip designs for stimulation follows, particularly for generation of programmable space–time patterns across a distribution of microchips.

## Circuit considerations for an RF powered microchip neural sensor/actuator

Several microelectronic designs have been published for wireless microchips, differing mainly whether applied to recording neural recording or neural stimulation (Yeon et al., 2018; Khalifa et al., 2019; Lee J. et al., 2021; review by Sahasrabudhe et al 2025). The first consideration in a sub-millimeter size chip as brain implant is the requirement of very low power. Simple estimates suggest that power on the microwatt scale is a necessary constraint dictated in turn by the maximum amount of RF power allowed by regulatory Specific Absorption Rates (SAR) of 1.6 W/kg brain mass for human use (Bailey et al., 2019).

For a neural recording microchip, its generic overall architecture can be divided into an analog front preamplifier, a digital controller, and circuits for energy harvesting and elementary. A perennial challenge in neural electronics in general here accentuated by requirements of ultra-low power and small footprint is the problem of designing low-noise preamplifiers.

In our labs we have experimented with various types of analogue front ends (AFE) frequently utilizing the “Harrison-type” (Harrison, 2002) ac-coupled transimpedance amplifiers. We have also experimented with a DC-coupled voltage-to-current converter merged with an analogue-to-digital converter via an area-efficient capacitor-less approach to achieve an input-referred noise of 2.2 μV 500 Hz bandwidth, occupying an overall footprint of 100 μm × 100 μm in recording at a 1 kHz sampling rate and 8-bit resolution (Huang et al., 2018). A DC-coupled AFE avoids bulky coupling capacitors but has a cost of a background induced DC offsets which can be particularly unpredictable in a dynamical body (tissue) environment.

As for neural stimulation, the footprint is smaller in the absence or amplifier circuits which typically require a sizable area though impedance matching circuits to the electrodes with tissue are required to optimize the current injection and caution exercised not to exceed the ‘water window’ electrolysis boundary. Small-size RF powered current injecting stimulators of size in the range of 300umx300um of silicon area (0.009 mm^3^ volume) have been reported (Khalifa et al., 2019; Lee et al., 2024a).

As one example, the block diagram composite of Figure 2 shows architecture of both a recording and a stimulating microchip design in the authors’ laboratories, fabricated in the TSMC 65 nm low power RF process (Lee J. et al., 2021). The upper half of the figure shows schematically how an ensemble of sub-millimeter size neurograins might be coupled wirelessly via an epicranial coil as part of a transcranial/transcutaneous inductive link for both power and digital telemetry (here near 915 MHz medical device band; a choice dictated by chip size and frequency dependent tissue losses). The epicranial coil, an umbrella whose area covers the entire multichip implant, is a necessary add-on in the all-RF approach especially in estimating system power demands. From point of implant surgery, the passive, biomaterial compatible epicranial coil need not be a burden, particularly if the polymer (PDMS) structure is dual used as a substrate for the microchip population.

**Figure 2:**
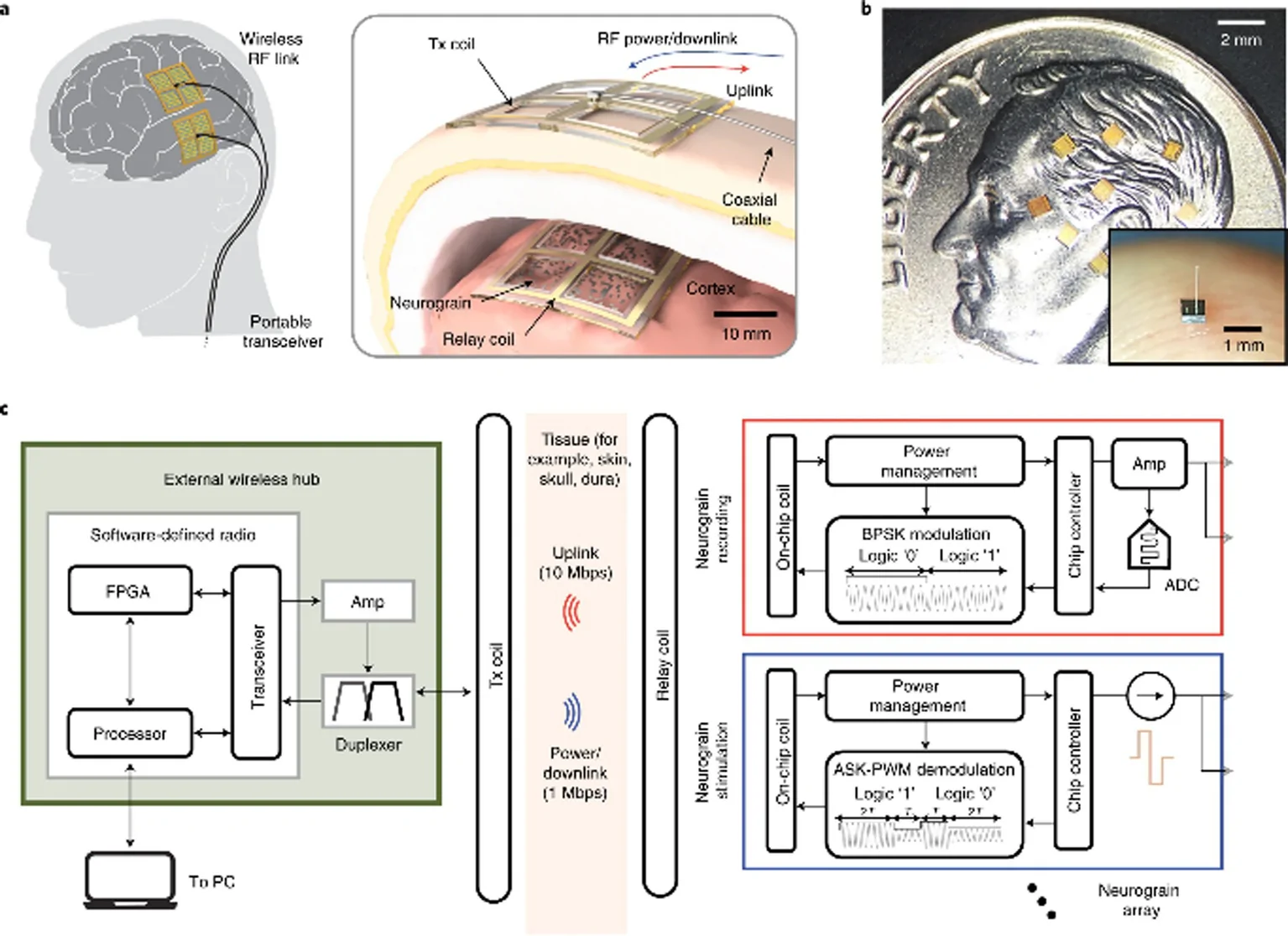
A prototype “neurograin” system for distributed autonomous network-based multipoing sensing or neural stimulation. **(a)** Concept of an implant where transcutaneous RF power and data link from single external antenna blankets an ensemble of neurograins; **(b)** Photo of sub-millimeter size microchips chiplets (the inset shows an optional post-process-integrated microwire for intracortical access); **(c)** Major elements of a microchip as part of a wireless network, consisting of the external RF hub (software defined radio transceiver), inductive coupling link and respective circuits for recording and stimulation. The common external hub deploys a field-programmable gate array (FPA) and a software designed transceiver (SDR) for the RF link; (from Lee J. et al., 2021).

The lower half of Figure 2 shows the principal circuit blocks for recording and stimulation by current pulse injection, as well as particular though not unique choices of digital RF modulation (BPSK and ASK-PWM) for transmission of neural signals (uplink) and for sending stimulation parameters (downlink). The digital circuits manage the on-chip data and serve a controlling function in this type of “system-on-chip” microchip. The design of the RF modulation scheme is discussed further below in considering methods for bandwidth sparse neural data transmission and generation of space–time patterned stimulation, respectively.

Neural data recorded by the on-chip pair of electrodes (planar or equipped by microwires) is received at the external software defined radio (SDR) by RF backscattering where the modulator block in Figure 3 toggles a tuning capacitor for binary phase-shift keying (BPSK). While ensuring that the magnitude of its input impedance maintains a constant value, the phase modulation is maximized by selecting the value of the toggling capacitor to maintain a stable power supply to the chip (Leung et al., 2018). Data are upconverted before backscattering using an on-chip, free-running relaxation oscillator with an operational frequency near 30 MHz or, alternatively, by acquiring a clock from frequency down-conversion from the external RF transceiver (Tx) itself. High-fidelity isolation of the backscattered data is quite important for low-noise demodulation and recovery at the external hub whether for online or offline analysis.

**Figure 3:**
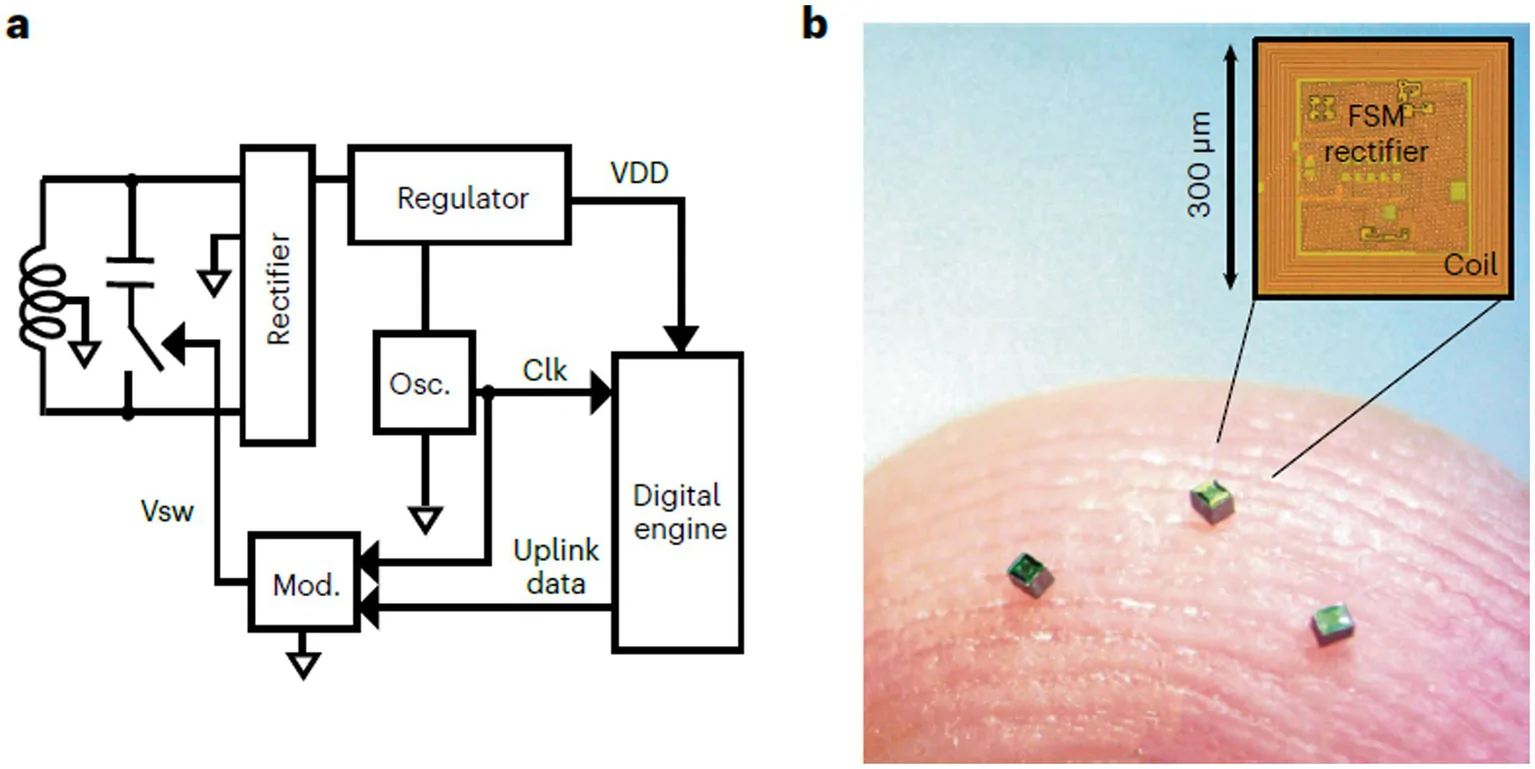
**(a)** Simple toggle circuit for RF backscattering of modulated RF neural data (downlink baseband 945 MHz; uplink backscattered signa at 915 MHz); **(b)** Size of a typical ‘neurograin’ (~ 300µmx300µmx100µm in size) (after Lee J. et al., 2021).

We note that both standard amplitude-shift keying (ASK), frequency-shift keying (FSK), load-shift keying (LSK) or use of infrared light in an ultrawideband modulation scheme have been proposed and used to communicate with a single or handful of microimplants although demonstrations have so far been limited to data rates below 1 Mbps (Yeon et al., 2018; Ha et al., 2016; Ahmadi et al., 2019). For large ensembles of spatially distributed microchips, a more versatile networking protocol with larger data bandwidth is needed as discussed later in this paper.

Regardless of the particular electromagnetic design for wireless power transfer, one critical circuit component on a microchip in any purely RF-dependent scheme is the choice of a rectifier block, impedance matched to the resonant LC-antenna. In early work we designed an efficient power-harvesting circuit feeding off a three-turn on-chip coil and utilizing a three-stage, low turn-on, deep n-well n-type metal–oxide–semiconductor transistor rectifier circuit with a low transistor threshold (Theilmann et al., 2012). Up to 48.5% rectification efficiency at −10 dBm input has been achieved through the avoidance of body effects in the n-type metal–oxide–semiconductor transistors in this design (Leung et al., 2018). At the component level, the rectifier efficiency compares well with, e.g., the photovoltaic converters on StiMote microchips (Lee et al., 2024b) and piezoelectric ultrasound Neural Dust.

## RF energy harvesting

Delivery of power to active biomedical implants without primary (embedded) batteries is a challenge that cuts across all these technologies. The problem is particularly acute for the types of sub-millimeter size microscale implants discussed here. So far there appears to be no viable means to harvest, e.g., energy directly from the body to power microchips, even if operating at sub-microwatt levels. Thus, wireless energy delivery remains the principal option.

A number of approaches have been taken for transcutaneous wireless power transfer (WPT) from energy sources outside the body. As mentioned, piezoelectric materials have been integrated into implants for transcranial energy harvesting and data transmission using ultrasound as the intermediary modality (e.g. Seo et al., 2015; review by Neely et al., 2018). Elsewhere, magnetoelectric materials were deployed in one class of neural implants (Chen et al., 2022). Optical techniques using infrared were proposed (Costello et al., 2022; Lee A. H. et al., 2021) and are attractive yet fundamentally hindered by losses from light scattering and absorption which increase rapidly with increasing tissue/bone thickness.

Power transfer by resonant inductive (near field) RF coupling is well-established with vast literature evidence of application across many technology sectors including macroscale medical implants (Van Schuylenbergh and Puers, 2009). Purely electromagnetic wireless power transfer (WPT) in the near field (inductive) has been subject of study by authors in millimeter-sized devices (Jia et al., 2019; Gutruf et al., 2018). As for far-field as well as the ‘midfield’ (Ho et al., 2013) regimes, several approaches have likewise been suggested for medical implants but are challenging when the target device (coil) size shrinks to millimeter and sub-millimeter scale. While achieving good electromagnetic coupling is, inherently challenging for implantable microchips (Ho et al., 2014), RF wireless power transfer (WPT) can improve by systematic antenna design. For example, sub-mm microdevices that provide wireless power for electrical recording and microstimulation in small animals was demonstrated (Khalifa et al., 2019; Yeon et al., 2018; Agrawal et al., 2017).

There is a major practical difference between an implant designed for recording vs. stimulation or neuromodulation by current delivery: Whereas recording of neural signals (for BCI and/or clinical monitoring) generally requires continuous powering, the case of stimulation benefits from lower duty cycle operation given the typical (though empirical) clinical values of ~100 μsec pulse duration at ~ 100 Hz repetition rate thereby reducing the average power demand.

In our laboratories, we have used the 915 MHz medical frequency band in pursuit of a practical WPT scheme for potentially very large ensembles of neurograins, the frequency based on several factors, including maximizing size and simple planar geometry of the on-chip microcoil, the area required by the on-chip impedance matching capacitor, the overall coverage area, and compatibility with semiconductor foundry (metal stacks) as well as postprocess microfabrication capability. The goal is a viable WPT scheme for implants built from large ensembles of microchips compatible with SAR limits to RF exposure in primates. Because of the thickness of tissue is expected to be at least 10 mm in humans, the addition of a ‘relay coil’ becomes necessary as already noted. Figure 4 shows a simple geometry where ‘window’ shaped (scalp external) Tx and a matching subcranial relay coil Rx form the dominant B-field energy pathway to funnel RF power to run an ensemble of cortical microchips, each chip equipped with its own on-chip < 300 um size microcoil.

**Figure 4:**
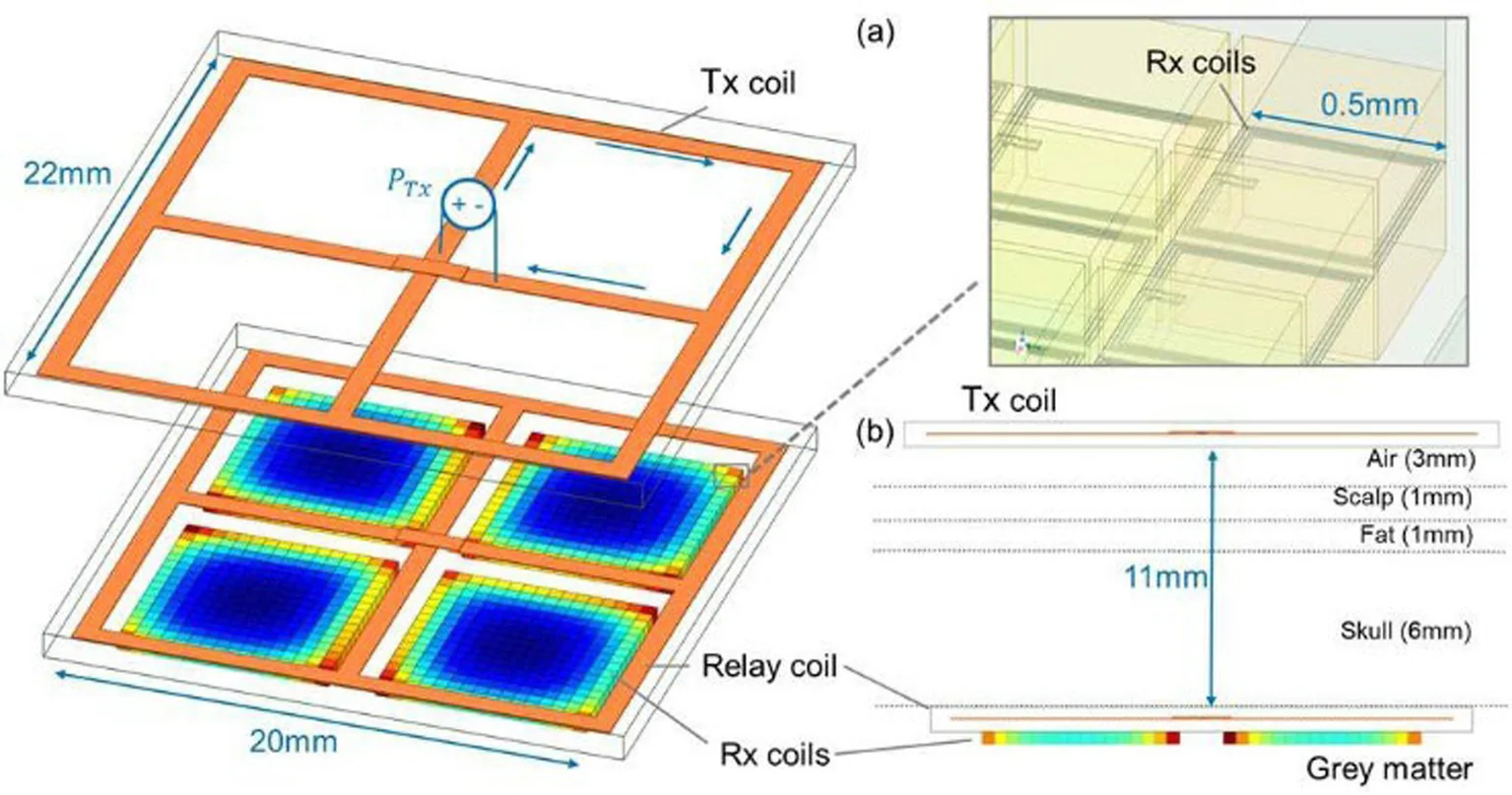
The concept of a relay coil: **(a)** 3-layer inductively coupled “Neurograin” WPT scheme. **(b)** Cross-section showing vertical distance between coils with respect to tissue layers for a non-human primate (after Lee J. et al., 2021; Daling et al., 2025).

The inductive powering arrangement in Figure 3 was implemented in rodent models in our early work (Lee J. et al., 2021). The four-quadrant thin flexible relay coil with copper traces embedded in PDMS insulation (Figure 4a) was placed on the epicortical plane co-locating the spatially distributed silicon microchips (microwires were added as intracortical probes to each chip in postprocessing, a feature not affecting the WPT). The quadrant shape coil maximizes wireless transfer across a targeted area of the cortex while minimizes interference from counterpropagating current flow. The heatmap of field energy in the relay coil shows the concentration near the edges of the conductor traces. Even so, electromagnetic simulations and measurements show that proper impedance matching is achievable to the load (i.e., microchip circuit) is effective in vivo experiments. The early work suggested that a microchip density up to of 250/cm^2^ (~ 1,024 individual neurograins in a 2 cm x 2 cm area) can also multiply the neural data transmission channel capacity without destructive interference in wireless data transfer. Supposing that each microchip requires a minimum power of approximately 25 μW (−16 dBm), a high value which needs to improve, one can estimate a wireless transfer efficiency of −40 dB as being necessary for chip operation at ~24 dBm transmitting power. This amount of Tx power challenges SAR limits and points to need for lowering chip power consumption perhaps and continued improvement in the electromagnetics WPT and associated antenna designs.

In addition to standard electromagnetic finite element (EM) simulations, the lumped circuit model of Figure 5 is useful in modeling the resonant three coil WPT for improving wireless power transfer (WPT) efficiency and reducing the specific absorption rate (SAR). Such circuit-based analysis is a standard tool in the broader literature of inductive coupling for wireless RF energy transfer whether for medical implants or cell phone charging. A model analysis assessed quantitatively the power delivered to the recording/stimulating nodes in the three-coil geometry, as well as power dissipated in the biological tissue layers (Alonzo et al., 2024). Surprisingly, the analysis suggested that lifting the external transmitter coil Tx slight above the scalp can beneficially reduce the SAR without undermining WPT efficiency.

**Figure 5:**
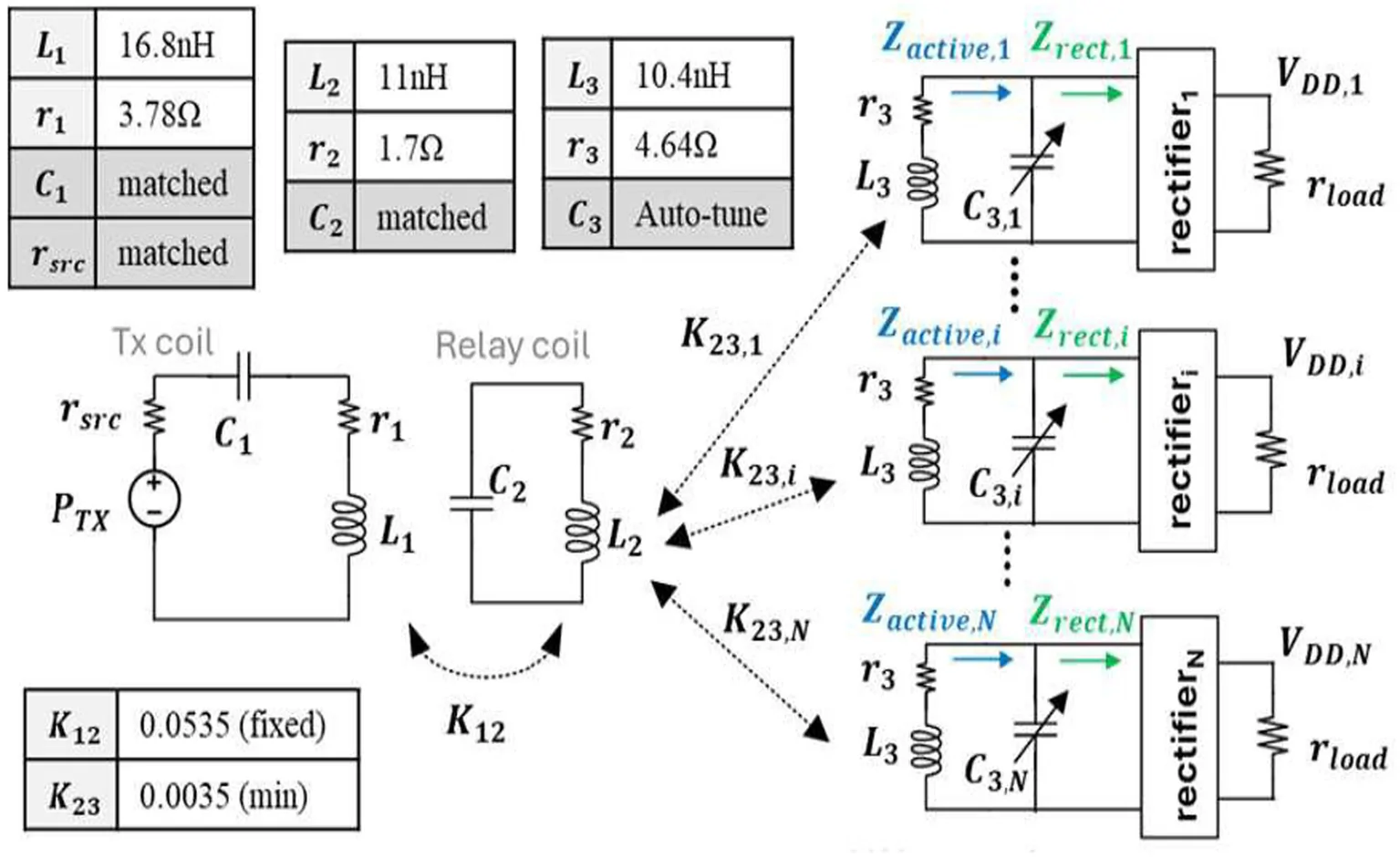
Equivalent lumped circuit model of the 3-layer resonant WPT system with representative values, identifying the spatially dependent coupling coefficients K_23,i_ for i = N neurograin implants with K12 fixed in one such simulation (from Alonzo et al., 2024).

One problem with the quadrant window design is that the B-field intensity is naturally higher around the edges of the thin film metal (copper) traces. Hence the impetus for designs to spread the field more uniformly while maintaining SAR performance compatible with values acceptable for primate use. An example of a recent idea to improve the field homogeneity is shown in Figure 6a by adding an L-shape piece of conductor to the relay coil to improve field homogeneity and, equally important, to reduce the ubiquitous Eddy currents resident in the Tx and Rx coils (Daling et al., 2025). In the simulation result of Figure 6b, the stored magnetic power in plotted in the ‘northeast’ quadrant of the shielded relay coil. Compared to Figure 2a, one can see how the high (excessive) power near the edges is removed. Figure 6c demonstrates the possible benefit of power re-distribution across the 256 microchip locations (16 × 16 array) when the shield is applied. The average stored magnetic power increases from 3.5 to 3.9 W/cm^3^ (by 10%) for the geometry while the standard deviation decreases from 1.8 to 1.2 W/cm^3^ (by 35%). The addition of the added metal L-shaped trace also suggests a benefit in reducing the SAR values (Daling et al., 2025). The experimental verification of these results awaits conformation at this writing.

**Figure 6:**
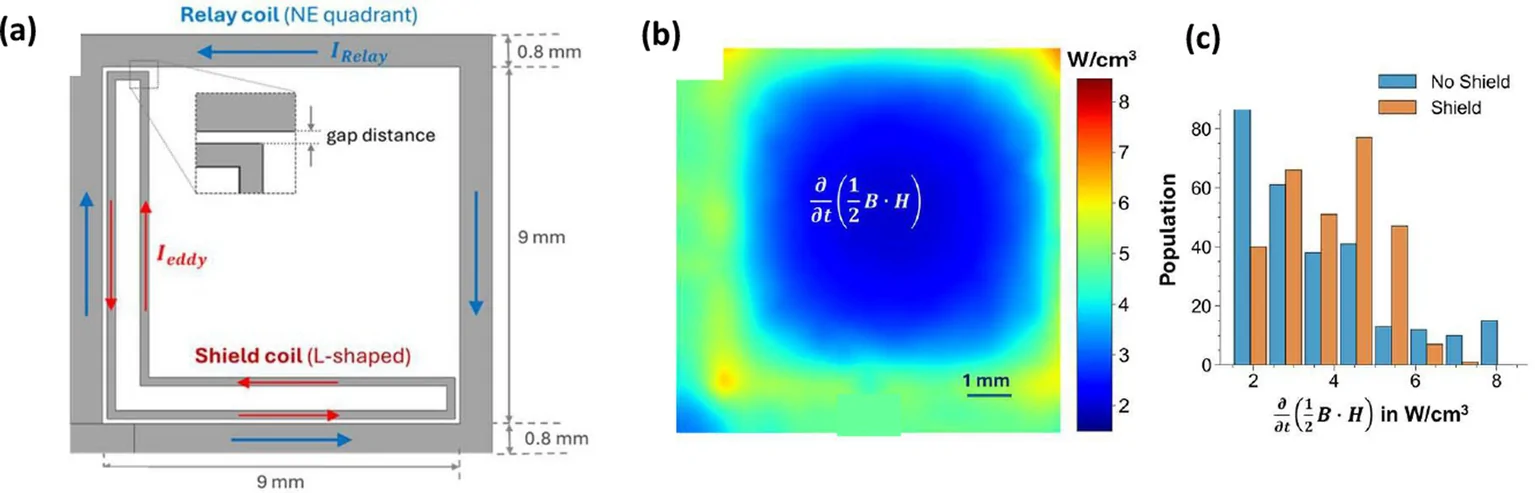
**(a)** Conceptual design of the shielded relay coil added to the ‘window coil’ with sample dimensions (‘Northeast’ quadrant); **(b)** Stored magnetic power of the shielded relay coil (NE quadrant); **(c)** Power distribution histograms with and without the additional shield (from Daling et al., 2025).

There is admittedly need and room for innovation to improve the overall RF-driven WPT transfer, homogeneity of the B-field distribution, hence spatial uniformity of WPT, and the SAR values acceptable for primate applications. Possibilities remain unexplored such as exploring other coil geometries (Jow and Ghovanloo, 2007), new high-permeability thin film magnetic materials to concentrate magnetic flux concentration at these relatively high frequencies where conventional ferrites lose their strength, potential of metamaterials (Kakaraparty et al., 2025) for enhancement from negative dielectric constants. Adding additional relay coils (RamRakhyani and Lazzi, 2013) offers additional design space through at a cost of surgical complexity; a four-coil system (Lee J. et al., 2021, supplement) could perhaps be realized by the pair of relay coils sandwiching the skull in a primate implant. There has been further research to explore expansion of other materials, nonplanar geometries, and adaptive circuit schemes (Kim et al., 2026).

## Wireless data transmission from large ensembles of distributed microsensors – what can brain connectivity teach us

Continuous and uninterrupted transmission of data wirelessly from large numbers of sensors, up to thousands and whether monolithic microelectrode arrays or independent recording nodes poses a nontrivial question about optimal communication strategy, balancing bandwidth, power, and error rates. The central engineering issue is that broadband spike recording creates very large raw-data rates potentially to Gbps range (typically for ~30 ksps channel sampling rates) and systems differ in terms of additional data compression and modulation approaches in their telecommunication protocols. In terms of macroscale BCI-targeting wireless implants, there are multiple suggested architectures most with data transfer protocols as adaptations of established digital telecommunication approaches. In terms of carrier spectrum these divide roughly to custom narrowband protocols tailored for wireless medical data transmission (Teshome et al., 2019) and ultrawideband impulse radio (UWB) (Lei et al., 2024). Examples of former are those using rapid multiplexing of signals across microelectrodes for serial broadband RF transmission (Min et al., 2013), sampling only a fraction of channels at high rate (Jung et al., 2025) or optical telemetry approaches (De Marcellis et al., 2020). A number of data compression strategies have been reported to reduce transmission bandwidth (Thorbergsson et al., 2014; Shaeri and Sodagar, 2015; He et al., 2024), including methods of manipulating the power spectrum (Irwin et al., 2016; Nason et al., 2020).

From communications engineering point of view, the case of spatially distributed autonomous, asynchronous microchip implants such as the neurograins is analogous to cellular networks with data streaming to a cell tower across user channels. The repertoire of digital modulation schemes includes basic binary phase-shift keying (BPSK) or schemes such as frequency-division multiple access (FDMA) and code-division multiple access (CDMA) developed to increase the channel transmission capability. In our earlier work exploring wireless scalability limits (Lee J. et al., 2021), we deployed a relatively power-efficient time-division multiple access (TDMA) network strategy for both communication and active management of system latency in a “call-and-response” mode. The approach, while offering power and size efficiency to the network, reached scalability limits for ~800 neurograins beyond which packet collisions elevated the error rate above an acceptable value ~10^−3^.

A brain science view attempts to emulate how neurons communicate (wired through axons and dendrites) across a cortical network using asynchronous action potential spikes and temporal spike trains to encode and transmit enormous amounts of information with superb energy efficiency. In our recent work, a neuromorphic event-sensing approach was explored for the spatially distributed neurograin system (Lee et al., 2024a). One key assumption in a neuromorphic approach is that events (as in spike neuronal firing rate) are sparse. Further, for the external transceiver hub to distinguish individual microchips streaming neural data from a large ensemble (thousands), encoding a unique device identifier (ID) on chip is necessary for the functional implementation of an addressable wireless network. We have successfully tested different ID approaches for recording neurograins: (1) a physical digital address, engraved on chip by laser ablation (Lee A. H. et al., 2024), and (2) an addressing scheme based on physically unclonable functions (PUF) (Yang et al., 2017; Suh and Devadas, 2007). As a postprocessing step the former is labor intensive while the latter exploits a PUF seeded encryption of addresses in a variant of code-division multiplexing (CDMA). As described in the next section, sending downlink commands for programming current parameters in a neural stimulation function requires a robust and fast addressing scheme for large populations of microdevices such as generating patterned cortical stimulation by wireless means (Lee A. H. et al., 2024).

In another analog, an ensemble of neurograins or other autonomous wireless microchip sensors using RF or optical techniques for wireless data transmission is akin to a distribution of RFID tags, the data from which is deciphered at once by a single reader. However, in contrast with readout methods used in the ubiquitous RFID technologies, here the ‘neural tags’ are active microelectronic chips and data dynamic across an ensemble. A single RF (or optical, acoustic) transceiver must the able to receive and decode the aggregate data from possibly thousands of microsensors where the signals from each (as well as their location), varies both in time and amplitude.

In adapting a neuromorphic approach for transmission of binary spike events, we designed a scheme (Figure 7) where the binary spike signals (events) are digitally encoded in a predesigned spreading code that contains a unique address for each chip. The chip itself is assumed an ‘event’ detector for neural spikes as well as a converter of dynamical analog events to trains of spikes. Each chip has an internal clock derived from frequency conversion from the baseband (here near 900 MHz) via the downlink, the approach offering the benefit of negligible clock variance and near-independence from energy harvesting efficiency (Man et al., 2007; Baghaei-Nejad et al., 2009; Hwang and Lin, 2009). The frequency divider approach does not imply network synchrony in this intrinsically asynchronous approach since the phases of clocks of individual chips will differ due to phase lags arising, for example.

**Figure 7:**
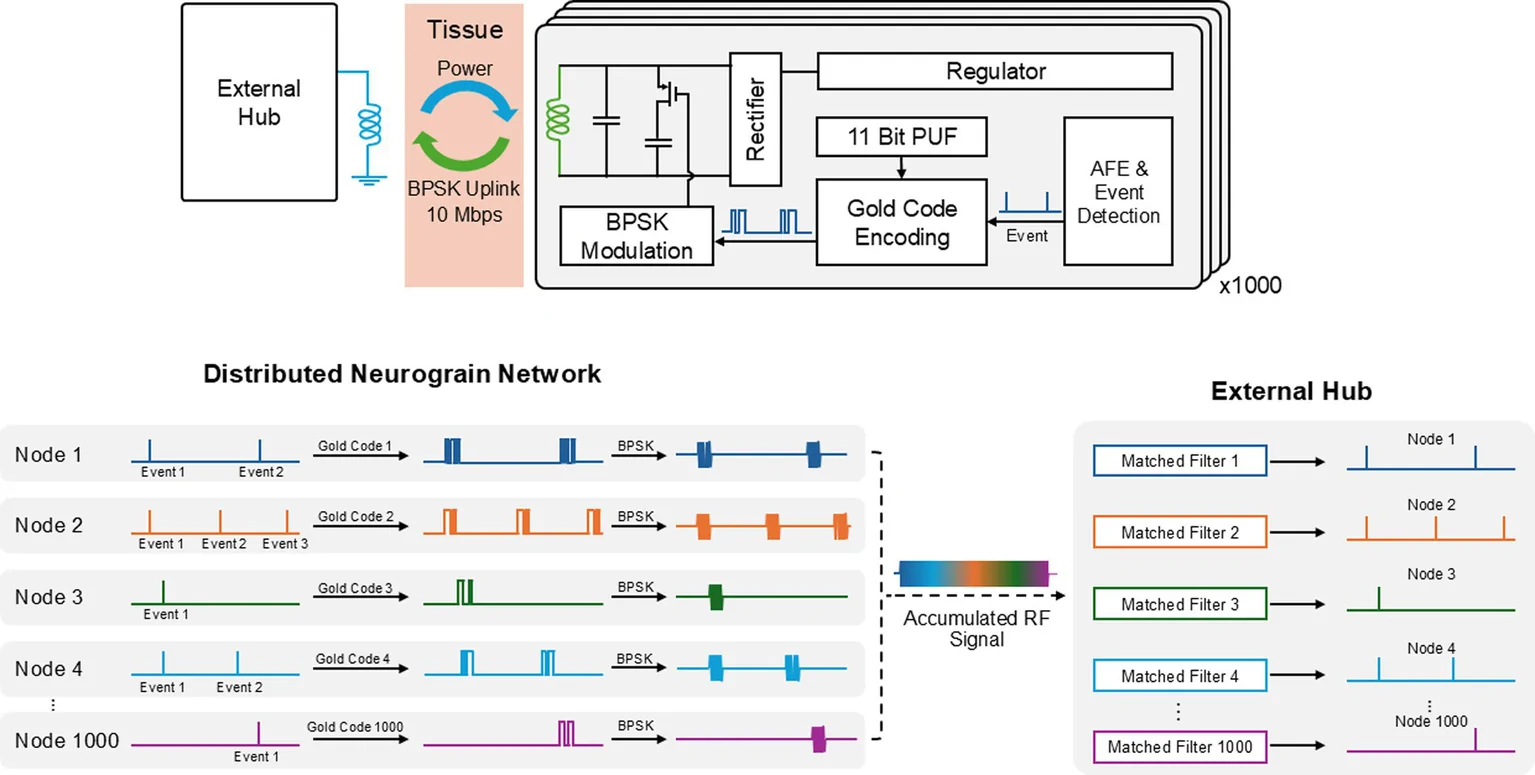
Principal circuit blocks of neuromorphic microchip designed for low-power, bandwidth efficient spike-based asynchronous communication (ASBIT); upper panel: principal circuit blocks onboard each microchip in a large ensemble of spatially distributed autonomous implants housing on-chip antenna, rectifier and digital logic circuits where the system-on-chip clock is downconverted by downlink from baseband; Lower panel: Schematic illustrations of the data stream in an ASBIT protocol and demodulation of the composite signal by matched filter set by post-transceiver electronics.

The idea that each microchip with a unique address is viewed as a spike-generating event sensor is has a counterpart in dynamical vision cameras (DVS) where each pixel (mimicking a retinal cell) reacts only to changes in illumination and encodes these changes as a spike train (albeit wired) output. Retina-emulating DVS cameras have emerged recently as candidates, e.g., for robotic vision (Liu and Delbruck, 2010; Indiveri and Douglas, 2000; AliAkbarpour et al., 2024). For wireless multichannel neural data we have developed a network protocol labeled as” asynchronous sparse binary identification transmission” (ASBIT) where data on each is multiplied by a unique pseudorandom number (PN), specifically the quasi-orthogonal Gold code, a case which has been theoretically analyzed for an RFID tag (Mutti and Floerkemeier, 2008; Yang et al., 2018). The code serves as the address for each asynchronous sensor node in an address event representation (AER) protocol (Culurciello et al., 2001), so that spike-event data at the external receiver specific to each chip can be recovered and unpacked such as by matched filters in Figure 7. A related idea involving an asynchronous AER approach specific to neural sensing applications by optical means was proposed in the optical communication domain by innovating a pulse interval modulation scheme (Costello et al., 2022). In this work computational simulations indicated the possibility of transmitting up to 1,000-channels of neuronal spike data using time-modulated light-emitting diode light (Costello et al., 2022).

A Gold code is a type of pseudo-random (PN) sequence used commonly in spread-spectrum communication. Here, chips encoded by a set of Gold codes permits neurograins in a network to share the same base frequency band without mutual interference while automatically filtering background signals from other nodes and background noise. Further, random semiconductor process variations in chip fabrication create a “physically unclonable function” (PUF) which we use as the seed for generating the Gold code as in the multiplication scheme of Figure 8. The PUF provides a random number unique to each microchip for the chip to synthesize its unique code. In practice, once chips arrive from foundry each PUF seed can be read individually by RF backscattering and stored. The cost in terms of the silicon area is minimal such as the 18-bit PUF and the Gold code generating, remotely programmable finite state machine (FSM) illustrated in Figure 9 where a linear-feedback shift register is combined with PUF to generate the Gold code sequence for a given chip.

**Figure 8:**
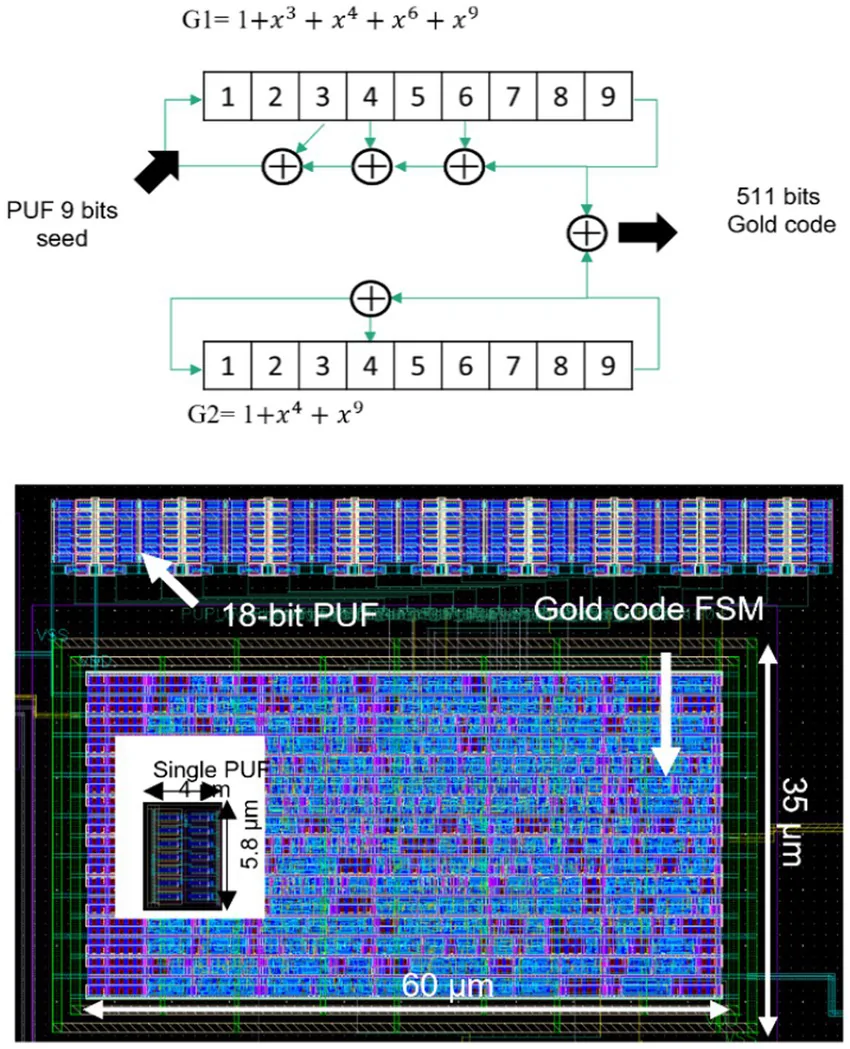
Implementation of the Gold code CDMA design at microchip level. Upper graphic: Schematic of a 511-bit Gold code generator, where a linear-feedback shift register is combined with a physically unclonable function (PUF) to generate the Gold code sequence; lower image: magnified view of the Gold code circuit as finite state machine (FSM) on a fabricated chip with a 35 μm × 60 μm footprint and the physically unclonable function (PUF) seed circuit with a unit size of 4 μm × 5.8 μm. The PUF provides a random number unique to each microchip for the chip to trigger the synthesis of a unique code as address for a chip. As an example, using 13 bits of the PUF as a seed sequence allows the synthesis a total of 8,191 bits of Gold code, even if only a portion of the Gold code may actually be transmitted in order to minimize the likelihood of PUF-induced collisions [statistical probability that same PUF sequence occurs in several chips (from Lee et al., 2024a)].

**Figure 9:**
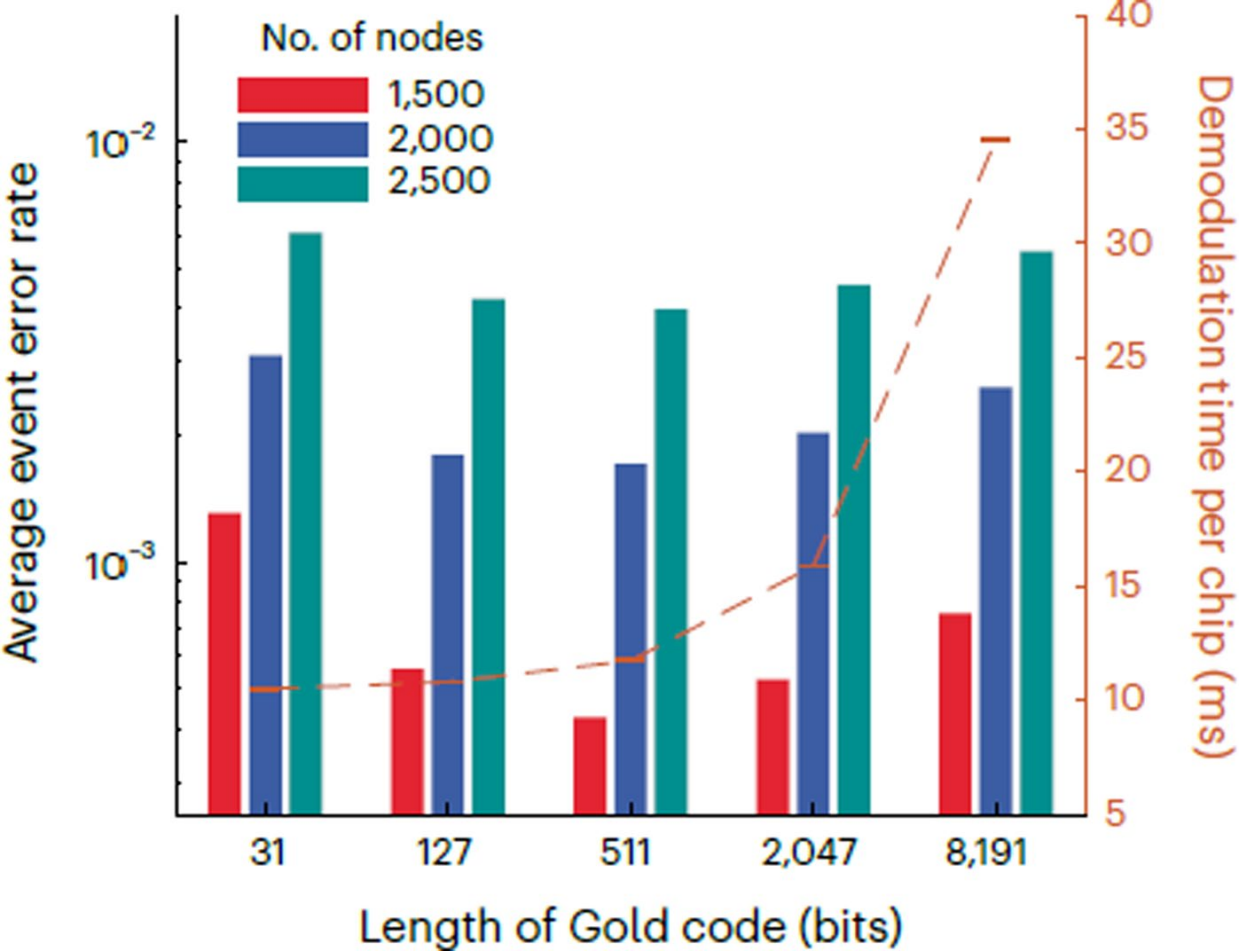
Exploring an optimal code length for network performance, here assuming a systemic signal-to-noise ratio (SNR) of 3.23 dB. The figure shows dependence of average event error rate (EER) on the number of nodes (microchips) in the network and the length of the Gold code. The plot also shows demodulation time per chip for a 1 s piece of data, showing the latency cost when increasing the length of the Gold code (here for an average event rate of 50 Hz and a transmission window of 6 s) (from Lee et al., 2024a).

At the software-defined radio transceiver (SDR) hub, matched filters can be used for event recovery by RF demodulation. In the process, the code sequence is first encoded using binary phase shift keying (BPSK) as a digital waveform by the on-chip clock (typically at 10 MHz). Data transmits via backscattering by a toggled capacitor in the transmission circuit on the chip (Figure 3). The generic SDR (here a module by “Raptor’ by Rincon Inc) down-converts the backscattered sideband to DC. Analyzing data from very large ensembles of microchips is computationally challenging, however, whereby performing demodulation such as by matched filter methods can make real-time operation challenging. However, the emergence of high-performance FPGAs can accelerate the computations to be compatible with low latency requirements by BCI systems.

A key metric for the performance of the event-sensing ASBIT wireless network is the error rate and its scaling to large ensembles of microchips. In this communication protocol, a bit ‘one’ represents an event detected at a given sensor node, accompanied by its Gold code. A useful way to quantify the fidelity of data transmission is to define an event error rate (EER) such that a missing event or an instance of false detection is an error. As an example, we assumed a nominal duration of each event (the bin size) of 1 ms, a choice relevant to spike detection such as in BCI applications. Figure 9 shows the average EER for 40 ‘target’ chips as a function of the number of other neurograins as their contribution to background interference and noise (i.e., all the nodes). The figure also plots the calculated EER with the length of the Gold code and downstream matched-filter demodulation time. Here, starting from a high event rate of 50 Hz, a value above an average cortical neuron firing rate the results of Figure 10 can be extrapolated to progressively lower event rates (sparsity) to estimate that the network can sustain up to 2,500 neurograins (nodes) with an acceptable EER (10^−3^) for BCI applications. The overall channel capacity of the network could perhaps be further increased by adding a finite amount of ‘edge’ computing capability on the microchips themselves, assuming that the increased complexity of architecture incurs only modest cost in power and footprint.

**Figure 10:**
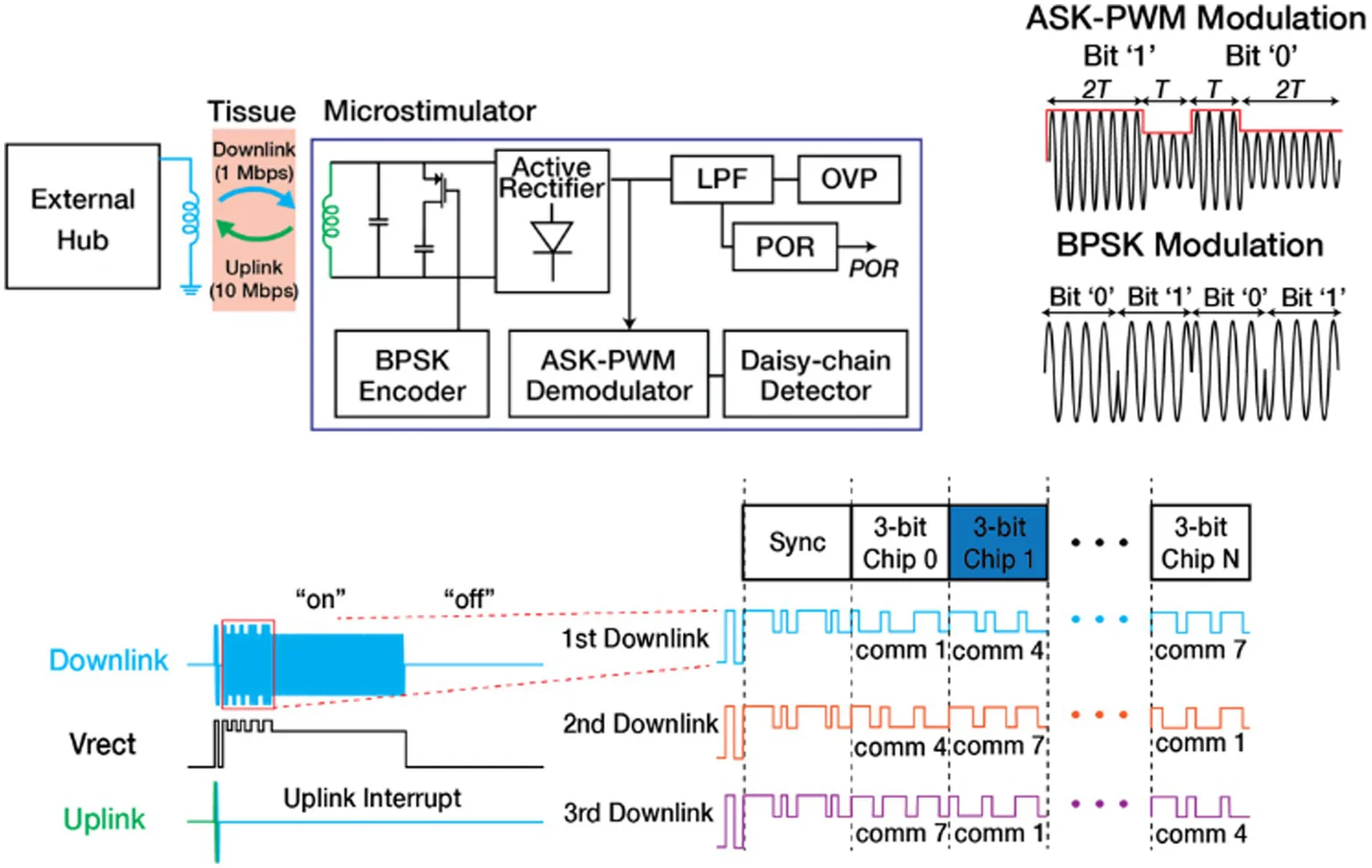
Microchip circuitry for a RF daisy-chain communication scheme for remote control of current stimulus. Downlink delivers RF power and commands (at 1Mbps data rate) to a microchip, to select one of the preprogrammed current waveforms. The circuit consists of blocks for power harvesting (active rectifier), data recovery by ASK-PWM demodulator, and the daisy-chain detector circuit. Power-on-reset triggers a BPSK uplink modulation circuit to report its unique address, driven by a free-running oscillator. Sample waveforms shown for the downlink and uplink. Bottom schematic illustrates how the downlink is ‘on’ for short periods to deliver RF energy to the chip when needed, resulting in low duty-cycled RF transmission lowering average RF exposure (Adapted from Lee A. H. et al., 2024).

## Space–time patterned stimulation by microchip ensembles

Monolithic intracortical or epicortical microelectrode arrays are suited to dual use in near-simultaneous recording and electrical or optical (Lee et al., 2016) stimulation. One area of active neuroscience research is the question of what space–time patterns of current stimuli might evoke naturalistic sensations and meaningful percepts. In recent BCI related research, human subjects report tactile sensations by patterns of current stimuli which target individual digits (Flesher et al., 2021; Valle et al., 2025). Improving understanding how patterns evoke visual imagery is the goal of several current projects in to develop viable visual cortical prostheses (Schmidt et al., 1996; Chen et al., 2020; Grani et al., 2022; Jiang et al., 2026).

While converting an MEA from recording to stimulation function is relatively simple from the sensor device point of view (say, changing higher impedance Pt electrode coatings to lower impedance IrO_x_ or conducting polymer coatings for example), there are many open and fundamental questions how electrical stimulation might serve as proxy for evoking a naturalistic sensation. In a sense-actuate dual mode, the main electronics challenge is the suppression of stimulus artefacts during of immediately after a current pulse.

In cases of wide area cortical stimulation, the ability to deliver programmable patterns of current stimuli over several cm2 by autonomous wireless microchips presents an opportunity to ‘break free’ from monolithic device platforms. In early work Khalifa reported the ‘Microbead’ as a miniaturized version of a silicon microchip aimed at stimulating the cortex or peripheral nerves by near field RF commands and suggested the use of multiple frequency bands to address individual microchips (Khalifa et al., 2019). The ultrasound-powered neural microstimulator was introduced for peripheral nerve stimulation (Piech et al., 2020) demonstrating the benefit of acoustic powering to reach deeper into tissue. Ultrasound also energized an endovascular neural stimulator housing a magnetoelastic film for energy delivery (Chen et al., 2020). In all the above cases only a single or few devices were implanted.

The capability to apply focused stimulation across the cortex by large ensembles of spatially distributed autonomous microchips poses a challenge to a scalable strategy where remotely controlled arbitrary time-amplitude distributed patterns excite selected targeted brain circuits. Each microchip injects currents locally according to instructions delivered through scalp and skull from an external energy source. As in the example of neural recording the chip may harvest its energy by RF coupling, now to convert captured energy into pulsed biphasic current output. We note that clinically established stimulation and neuromodulation protocols operate at low duty cycles. In an average case, biphasic current pulses ~100 μsec in duration are injected at ~100 Hz repetition rate, a duty cycle of 10^−2^. A low duty cycle lowers the average RF power and is advantageous in principle, in allowing higher peak current amplitudes while maintaining the system below regulatory SAR limits.

An approach to multi-chip communication protocol with downlink/uplink digital modulation schemes for ensembles of stimulating microchips is summarized in Figure 10 (Lee A. H. et al., 2024). Here harvested RF power converts into pulsed biphasic currents of up to 120 μA peak-to-peak in amplitude. For delivery of intracortical currents, pairs of tungsten microwires separated by 100 μm could be additionally wirebonded onto pads of epicortical silicon chips as a post-process step such as using conductive epoxy plus non-conductive shield encapsulation.

The RF communication scheme aimed to program the relevant stimulation parameters (current amplitude, period per phase, and repetition rate) by devising a protocol suitable for scaling to a large network of spatially distributed chips in real time. We developed a custom collision-free low duty- cycle communication protocol, a wireless register-mapping method denoted as a ‘daisy-chain’ protocol. The approach allows the control of the full network of microstimulators to generate space–time patterned stimulation only during the delivery of an actual pulsed current stimulus to reduce the total average RF power exposure.

An external RF transmitter (SDR transceiver) issues pre-scheduled commands across the chip population inside the area defined by the size of the epidermal and relay coils via a highspeed downlink at 1 Mbps. A particularly useful encoding choice is the amplitude shift keying pulse width modulation method (ASK-PWM). In an ASK-PWM scheme, the relative durations of high and low amplitude RF signals encode bits 1 and 0 at a data rate of 1Mbps as illustrated in the bottom half of Figure 10. This has the benefit that on-chip downlink bit recovery is tolerant to variations in the frequency of the on-chip free-running oscillator. The on-chip ASK-PWM demodulator decodes the envelope of the rectified incoming RF signal to extract the significant downlink bits, a string which is analyzed by the daisy-chain detector circuitry. The downlink bit stream consists of a ‘sync’ sequence followed by serialized commands for the full ensemble of chips without yet requiring any chip address information or enable sequence. Once a correct ‘sync’ sequence is detected, the circuit identifies the N-th command (three bits per chip) assigned to a specific chip with its unique address N. The address, unique to each chip, can be predefined by laser-ablating metal fuses in a chip postprocessing step (Lee et al., 2023). A three-bit downlink instruction can be delivered in mere 3 μs to program a specific stimulation waveform for an individual chip; hence, in principle, arbitrary space–time patterns of microstimulation can be generated by the scheme for up to a thousand chips within 3 ms latency. In this version, the digital controller uses the three-bit command from the daisy chain detector to configure the stimulation waveform generated by the current source driver (example in Figure 11), adjusting amplitude, phase duration, and pulse repetition rate. A programmable operational amplifier current source driver delivers a predefined current across the tissue, alternating between the cathode and anode to minimize detrimental net charge accumulation effects. A feedback loop with a reference voltage (V_ref_) ensures consistency in the current delivery to target tissue and is immune to impedance value at the electrodes.

**Figure 11:**
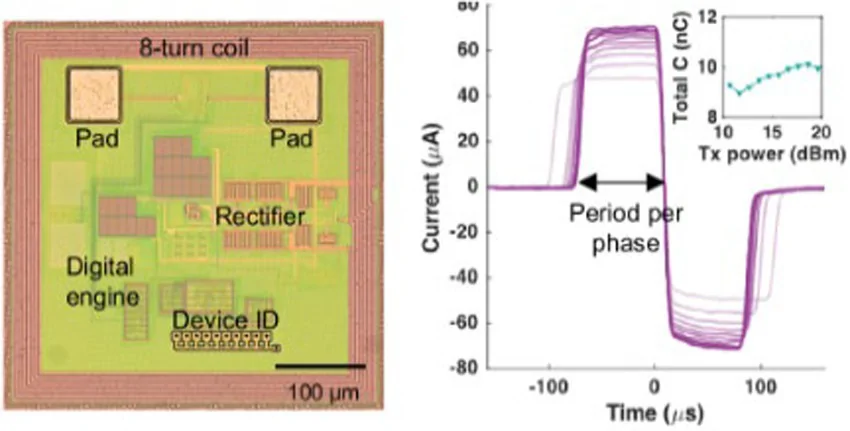
Left: Microphotograph of a 400 μm × 400 μm stimulator chip. Right: The current waveform for a 200 μs long pulse (100 μs per phase) at varying T_x_ power levels. The inset shows the modest dependence of total injected charge on T_x_ power (from Lee A. H. et al., 2024).

A first step in assessing the performance of an ensemble of microstimulators prior to implant into an animal is a benchtop immersion test to a conductive tissue proxy phantom such as buffered saline. Based on each specific downlink command, varying amounts of charge are delivered by the chips as shown in the bottom row data of Figure 12b. While a finite charge imbalance remains, this amount is small relative to the total injected charge (generally less than 2%) because of active charge balancing. A custom(wired) 24-channel planar recording electrode (inset of Figure 12a) was used to map the arbitrary spatiotemporal patterns by a 24-element wireless network of microstimulators, here dynamically writing out a series of letters spelling ‘BROWN’ in spatiotemporal sequence (Figure 12c).

**Figure 12:**
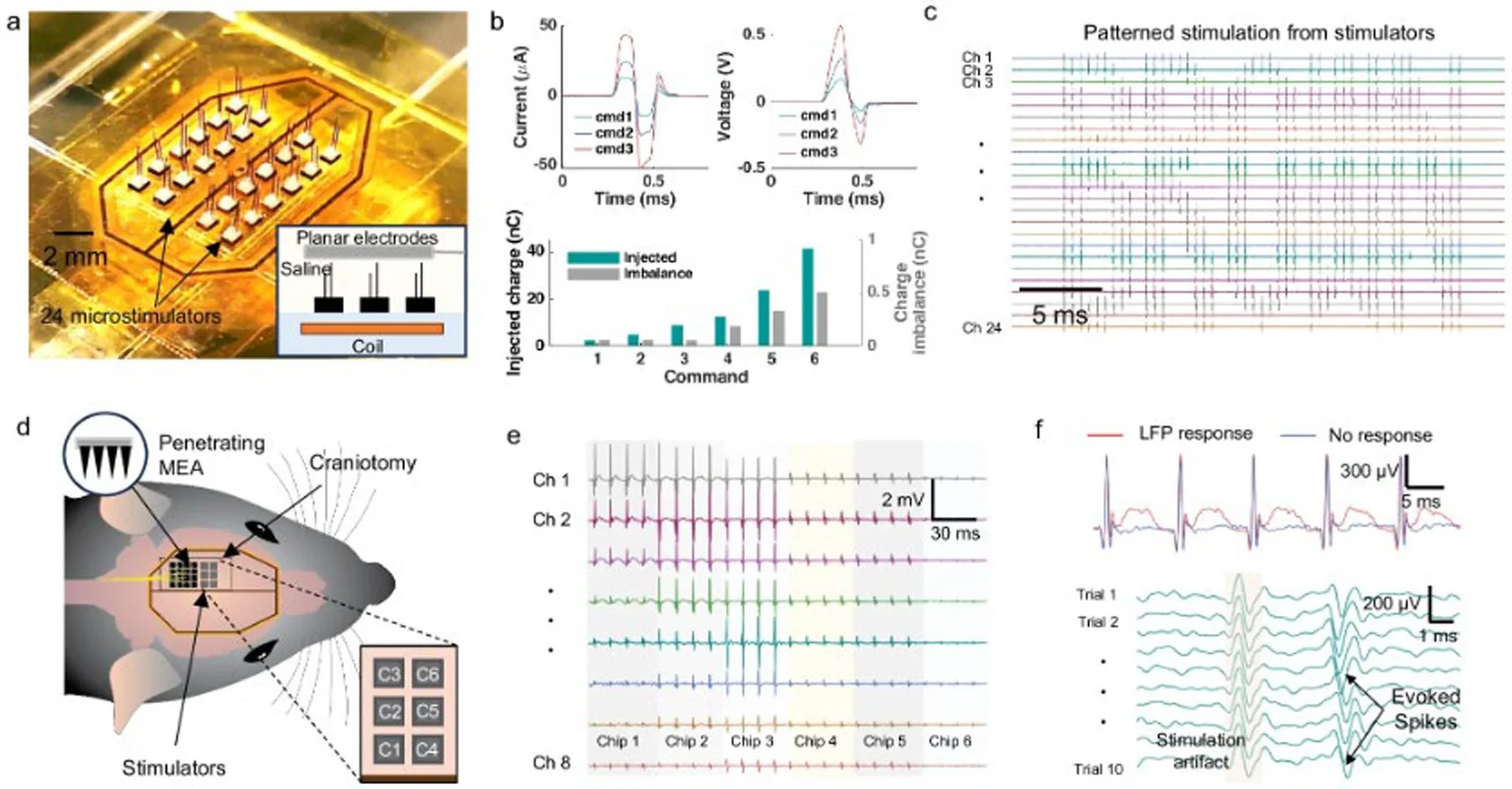
Demonstration of pattern stimulation by the wireless stimulator network in saline and in-vivo acute rat model. **(a)** Photo on microchips immersed in saline. The inset shows the location of the relay coil (bottom of the dish) and a 24-element planar MEA recording array; **(b)** Voltage and current waveforms injected by several microchips into the saline (top row). The bottom row shows injected total charge and charge imbalance and dependence on stimulation waveforms; **(c)** Spatiotemporal waveforms by an ensemble of 24 microchips to generate a current pattern spelling ‘BROWN’; **(d)** Microchips in rat cortex for acute experiments showing spatial location of six chips (C1 to C6) and the adjacent probe MEA with electrode separation of 400 μm; **(e)** A plot of the ‘stimulation artifact’ by each of the 6 chips as recorded across 8 channels of the MEA; **(f)** Cortical LFP responses evoked by such microstimulation (red, top); absence of response is observed through a given after repeated stimulation suggesting apparent neuronal fatigue (blue, top). The plot at the bottom displays the evoked neuronal spike responses following each trial (stimulation event). (Lee A. H. et al., 2024).

Up to three dozen silicon microchips, each as small as 300umx300um (100um thickness) were first implanted to the cortex of an acute rat (Figure 12d). For short term hermeticity chips were sealed by 1–2 μm thick envelope of parylene (except at electrode sites). A pair of 1.5 mm long tungsten microwires for intracortical penetration attached by conductive epoxy onto the electrode pads although more sophisticated automation of the microwire-to-chip bond will be required in large scale postprocessing in the future. Note that the proximity of the microwire pairs concentrates current density on 100 μm spatial scale. Integrating an auxiliary multielectrode recording array into the experiment, we could map both the excitation as well the neural response as shown in the matrix of data in Figures 12d,e,f.

The neurograin paradigm for multipoint intracortical microstimulation was demonstrated in chronic in-vivo rat model where a small group of six chips were implanted into a freely moving rat across sensory and motor areas for three months. Rats were trained in lever-pressing tasks and behavioral indicators/metrics used to monitor the outcome of neuromodulation experiments such as motion tracking imaging cameras. While more details can be found in Lee A. H. et al. (2024), we show here an example of resonant-like modulation of whisker movement due to stimulation at high frequency of 400 Hz he evoked response was also measured as post-stimulus changes in the whisker angle (Figure 13). Postmortem observation (by micro-X-ray scans) showed that the location of the implanted microstimulators remained stable over the period of 3 months, possibly aided by the microwire electrodes anchoring the free-floating epicortical chips. Likewise, there was no detectable indication if foreign-body tissue reaction in immunostaining studies. However, these studies did not address the remaining questions regarding brain micromotion, shear forces, nor long term chronic foreign body tissue response. Some of these issues have been studied in recent work on dummy silicon motes integrating intracortical carbon microfibers in the rat model (Letner et al., 2026).

**Figure 13:**
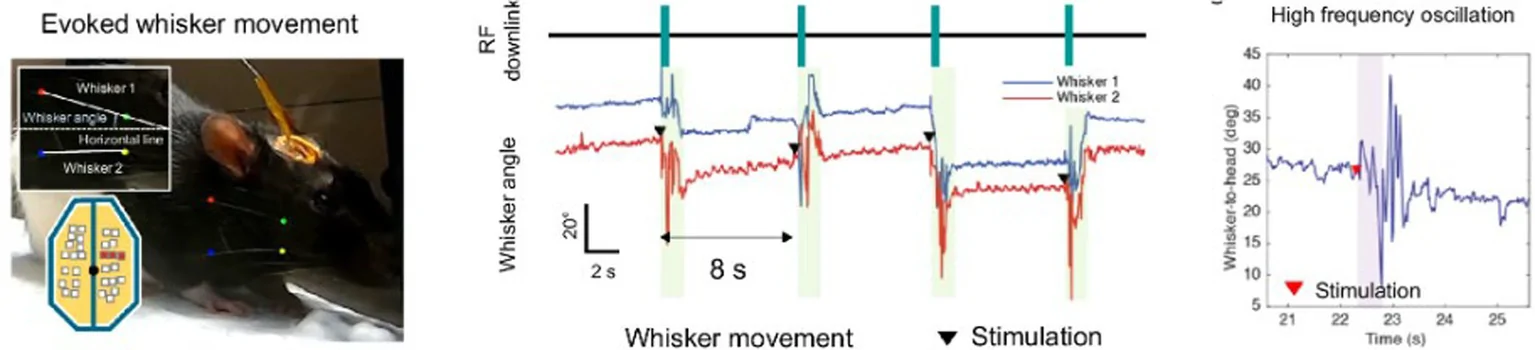
Left: Head shot of the animal with two target whiskers identified by dot markers as position indicators. The inset shows the location of three particular microchips (each injecting pulsed current at the high frequency of 400 Hz, 500 μs per phase, and 500 ms train duration); Center: Timing of the RF commands and stimulus-evoked whisker movement; whisker angles are measured with respect to the horizon (red and blue, respectively); Right: High-frequency oscillatory motion of evoked whisker response induced by the current stimulus into whisker area of the cortex. The shaded area on the graph represents the duration of the neuromodulating pulse train (from Lee A. H. et al., 2024).

Beyond opportunities for tactile, auditory, and visual prosthesis, the concept of a wireless network of spatially distributed microstimulator might offer an alternative to monolithic implants in varied restorative and therapeutic scenarios such auditory cortical ‘hearing aids’, large area cardiac stimulators and others that can benefit from distributed multipoint, programmed patterned electrical stimulation. Elsewhere, the ability to deliver meaningful restorative commands, e.g., to damaged spinal cord and peripheral nerves is an opportunity though pulsed currents in the milliampere range are probably required. This could be feasible by modifying the current driver and energy harvesting blocks of the microchip circuits described above, at a cost to the footprint of the RF coils and chips themselves - while still capitalizing from an inherently power efficient, low-duty-cycle system-level approach.

## Hermetic encapsulation of microchip implants

Proper hermetic encapsulation of microscale active electronic implants is non-trivial. Typically, in the case of wireless, macroscale brain implants of several cm^3^ in size in terms of their electronics payload, the payload physically separated as a hermetically sealed module connected by wire through hermetic feedthroughs to the microelectrode probes. On such size scale, established manufacturable hermetic packaging techniques such as Ti-enclosures (Borton et al., 2013) with pedigree derived from pacemakers or, more recently, ceramic enclosures (review by Shen and Maharbiz, 2021) offer a robust solution but one not easily applicable to sub-mm^3^ size packaging. Of most common dielectric thin film materials, Parylene-C, SiC, SiO₂, Al₂O₃, HfO₂, diamond-like carbon, or multilayer stacks thereof deposited directly over the electronics are making progress for hermetic sealing of microchips made but have not demonstrated the same decades-long hermeticity as conventional metal/ceramic packages. Achieving a reliable, conformal seal is particularly important at mechanically weaker points such as corners and edges of a diced silicon chiplet. An interesting approach for hermetically enclosing implant electronics at a wafer- or chip-scale is the use of narrow Au: Sn eutectic seal rings with the addition of in-silicon feedthroughs is highly desirable (Szostak et al., 2021). A general review of compatible biomaterials including a range of deposition techniques can be found in Yang et al. (2021).

In our laboratories, we have explored the use of atomic layer deposition (ALD) of multilayers of ultrathin SiO_2_ and HfO_2_ layers (Jeong et al., 2019). The ALD approach offers conformal coating *en masse*, deposition temperatures acceptable to microchip circuits of below 300C, and a biocompatible sub-micrometer thin encapsulant which adds only fractionally to the overall volume of a microchip implant. Multilayer, few atom layer thin layers were stacked to ~100 nm overall thickness, the barrier design intended to prevent ingression of water (HfO_2_) and ions (SiO_2_), respectively, while maintaining electromagnetic transparency necessary for wireless powering and RF communication near the 1 GHz frequency range. Accelerated lifetime testing on benchtop of chips immersed in hot saline (~80C) were used to extrapolate an expected lifetime up to 10 years, a promising result waiting for further validation *in vivo* animal models. Thus, for human use, a definitive solution to the hermicity problem for packaging of sub-mm size microchips for long term chronic use is still a fully unmet challenge both in terms of thin film materials and manufacturable microfabrication methods. Recently, multiyear stability of silicone (PDMS) encapsulated larger CMOS chips (Idil et al., 2025).

### Implantation techniques

Multiple factors must be considered in the implantation of novel sensors to brain tissue. These include minimizing tissue damage or trauma upon insertion, effects of long-term immune reaction by presence of foreign body materials, and safety and efficacy matching surgical protocols in the operating room. Established and clinically approved methods for silicon microelectrode arrays include pneumatic insertion technique of the Utah array (Rousche and Normann, 1992) and in case of carrier-supported flexible threads sophisticated robotic insertion means (Musk and Neuralink, 2019). A less invasive approach was tested in porcine and cadavers to insert an integrated active silicon ultrathin wafer implant via a slit incision (Hettick et al., 2026).

Two early examples in exploring methods for implanting large numbers of microchips using passive proxy ~100 um sized microparticles studied for large scale insertion/injection include the use of bioresorbable polyethylene glycol carrier (Sigurdsson et al., 2020) and a more thorough study of the bioresorbable approach by which batches of proxy wireless motes with carbon fiber electrodes were implanted into rat cortex (Letner et al., 2026). Surgically viable methods for implanting, say a thousand wireless microchips within requirements imposed by surgical constraints are yet to be developed. Possible automatic alternatives might exploit robotically assisted pick-and-place equipment such as in semiconductor chip industry perhaps in conjunction with bioresorbable carriers of ensembles of microchips.

## Brief comment on decoding large scale neural data - computational challenges

In an assistive BCI system, captured data is signal processed for input to computing machinery dedicated to neural decoding. Multichannel neural decoding by machine learning algorithms has become choice tools of computational methods especially when dealing with recordings from thousands of neurons, reviewed, (e.g., in Glaser et al., 2020; Livezey and Glaser, 2021).

In anticipation of the need for more sophisticated next generation of BCI technologies, a looming decoding challenge merging from increased channel counts (numbers of microelectrodes or microchips) in implants for large scale intracortical recording. Thousands of channels, when decoded simultaneously and real time, pose a challenge both to computational models and computing power. In context of envisioning the ‘neurograin-type’ microchip fully wireless ensembles of this article, we note that the ASBIT brain inspired spike-based event sensor network protocol described in this article can help solve at least part of the wireless data transfer problem by reducing the bandwidth and providing low latency transmission. If the individual sensors and network-based data transmission both use spikes and spike trains as fundamental bits of information, a computational approach which likewise uses spikes in implementing decoding strategies may offer advantages over other artificial neural network-based machine learning approaches. This suggests that examination of spiking neural networks (SNN), a rapidly expanding field neuromorphic computing offers new opportunities to large sale decoding. We note that (outside neuroscience) directly trained deep SNNs have recently become computationally practical through surrogate-gradient learning (Neftci et al., 2019; Eshraghian et al., 2023; Fang et al., 2023) and spike-driven transformers now approach ANN accuracy while retaining sparse, addition-only computation (Zhou et al., 2024; Yao et al., 2024).

Early ideas have been explored via simulations at our laboratories in the joint context of scalability of an end-to-end, event-based wireless BCI. In conjunction with a modest SNN model, we used open- source non-human primate data recorded from the primary motor cortex (M1) and its mapping on the somatosensory cortex (S1) (Fagg et al., 2009; Benjamin et al., 2018), both associated with hand movement intentions in a cursor-control task, to synthetically scale hypothetical spiking data to thousands of spiking neurons through simulations. Figure 14 shows a schematic flow of spiking neural data from a population of hypothetical wireless microsensors through an end-to-end spiking sensor network. The SNN decoder, here a modest three dense neural network layers and a sigma–delta neuron input and output layer used binary spike data to generate a reconstructed cursor velocity (Ghosh-Dastidar and Adeli, 2009; Orchard et al., 2015; Dethier et al., 2013; Kumarasinghe et al., 2021). The objective of the exercise was to simulate a large-scale wireless system the open source data was used to synthesize equivalent spiking data from 8,200 separate nodes (neurograins). Inclusion of the wireless transmission is important to assess the contribution of stochastic transmission noise. Figure 15 compares the SNN-model decoded and predicted hand velocity along the x-coordinate without transmission noise (wired system with S/N = 0) and the ASBIT wireless system. We also note recent work where a recurrent SNN-model was implemented in real time on a microcontroller hardware using primate microelectrode array-based motor data (Muten et al., 2025).

**Figure 14:**
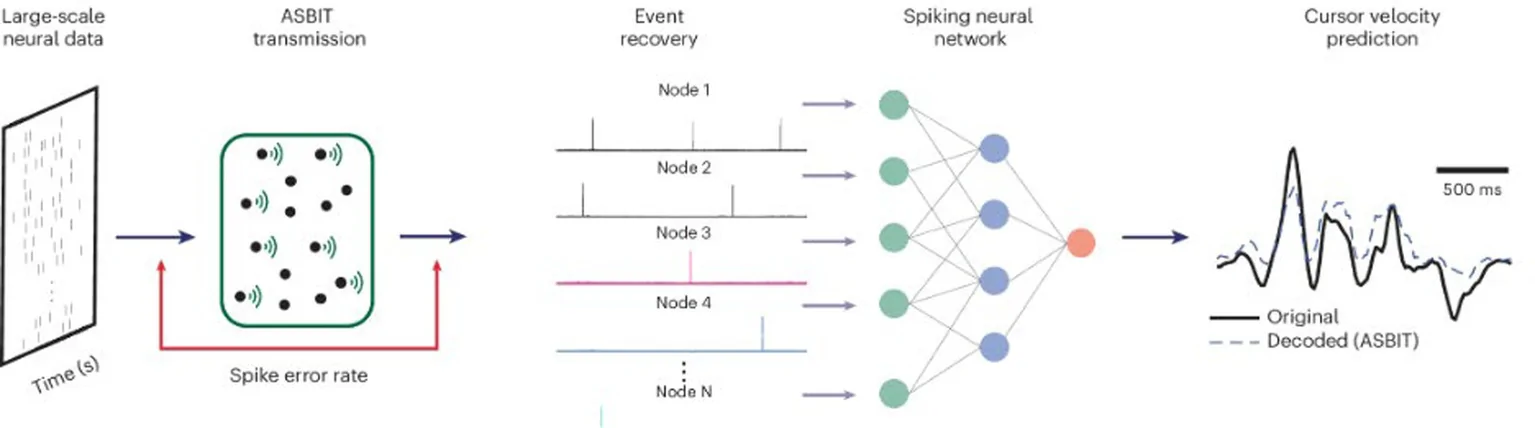
Schematic illustration of the wireless transmission of neuronal spike events using the ASBIT protocol. Received signal at an external wireless hub demodulates to extract spike data from each neurograin node as input to an SNN-based decoder to predict cursor velocity (from Lee et al., 2024a).

**Figure 15:**
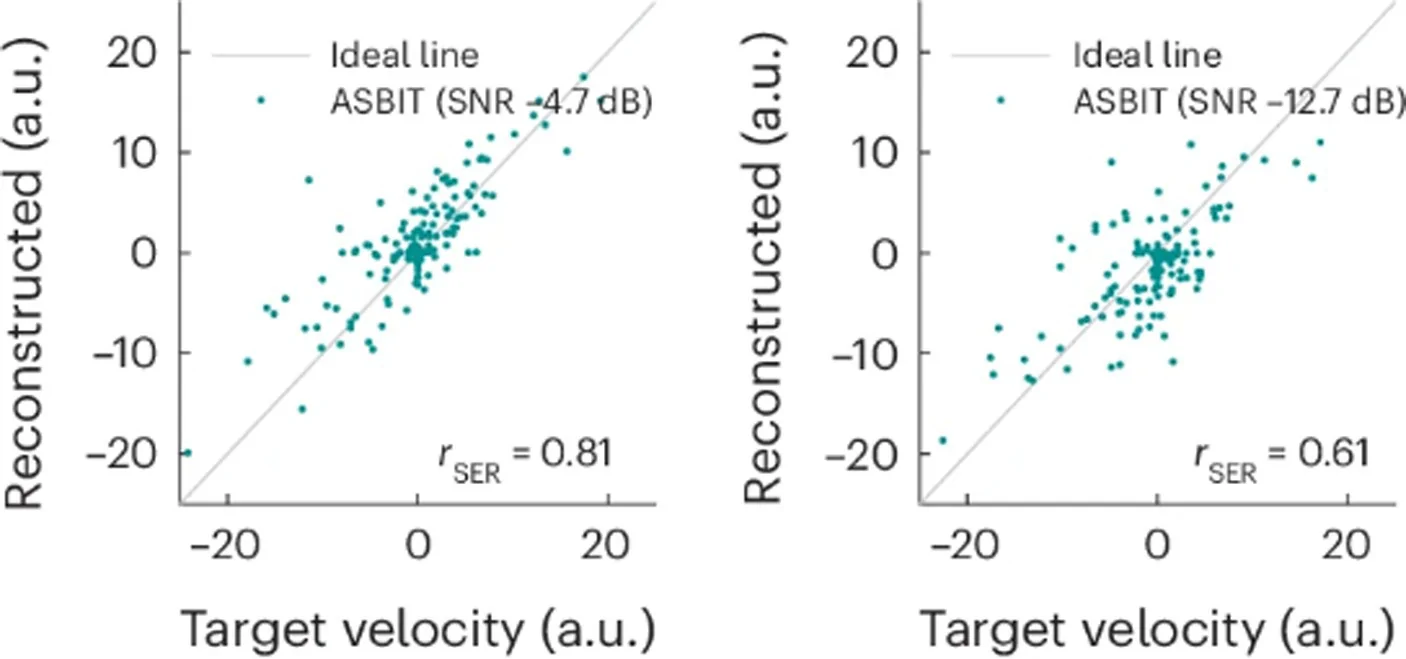
Kinematic target x velocity and reconstructed x velocity from the SNN neural decoder. The ‘ideal’ line assumes a noiseless wired recording system compared with decoder output using spike data transmitted through the wireless ASBIT protocol from 8,200 simulated nodes (hypothetical neurograins) for two cases of a different SNR (from Lee et al., 2024a).

## Summary

This article has focused on recent progress in the development of microscale sensors and actuators (stimulators) as one approach to next generation of brain-computer interfaces. Design and fabrication of sub-millimeter sized silicon chiplets as well as wireless network communication strategies have been illustrated by examples. A brain inspired idea of sparse, asynchronous event-based communication scheme for transmitting neural data was described as a bandwidth and power efficient method for implementing a low latency network capable of operating at low error rates. As for neuromodulation by current injection such as for intracortical stimulation, a method for generating arbitrary space–time excitation patterns across the cortex via a daisy-chain command-and-control network protocol was described.

As for future, combining both the recording and stimulation functions on a single implantable sub-millimeter size chip would offer a path to fully closed-loop wireless BCIs, to collectively record from and stimulate brain circuits by up to thousands of spatially distributed wireless microsensors. A network of such dual-function microelectronic chips will communicate bidirectionally with an external command center, enabled by RF network design which must now be agile and adaptive. The requirements of high-data rate, low-error rate, and low latency require innovative hardware and software solutions. The inclusion of a low-latency, power efficient neuromorphic machine learning platform to close the BCI loop through deep spiking network custom algorithms might allow the network to manage system resources. The work summed in this article has been motivated help to advance technology development for severely disabled people, particularly toward lightweight mobile systems as sensory and neuromotor prostheses in the central nervous system.
